# LncRNA KCNQ1OT1 (potassium voltage-gated channel subfamily Q member 1 opposite strand/antisense transcript 1) aggravates acute kidney injury by activating p38/NF-κB pathway via miR-212-3p/MAPK1 (mitogen-activated protein kinase 1) axis in sepsis

**DOI:** 10.1080/21655979.2021.2005987

**Published:** 2021-12-03

**Authors:** Haixia Wang, Hongbin Mou, Xiaolan Xu, Changhua Liu, Gang Zhou, Bo Gao

**Affiliations:** a Department of Critical Care Medicine, Subei People’s Hospital of Jiangsu Province, Yangzhou, China; b Department of Nephrology, Subei People’s Hospital of Jiangsu Province, Yangzhou, China

**Keywords:** Acute kidney injury, MAPK1, KCNQ1OT, p38/nf-κB, ceRNA

## Abstract

Acute kidney injury (AKI), a common complication of sepsis, is characterized by a rapid loss of renal excretory function. A variety of etiologies and pathophysiological processes may contribute to AKI. Previously, mitogen-activated protein kinase 1 (MAPK1) was reported to regulate cellular processes in various sepsis-associated diseases. The current study aimed to further explore the biological function and regulatory mechanism of MAPK1 in sepsis-induced AKI. In our study, MAPK1 exhibited high expression in the serum of AKI patients. Functionally, knockdown of MAPK1 suppressed inflammatory response, cell apoptosis in response of lipopolysaccharide (LPS) induction in HK-2 cells. Moreover, MAPK1 deficiency alleviated renal inflammation, renal dysfunction, and renal injury *in vivo*. Mechanistically, MAPK1 could activate the downstream p38/NF-κB pathway. Moreover, long noncoding RNA potassium voltage-gated channel subfamily Q member 1 opposite strand/antisense transcript 1 (KCNQ1OT1) was identified to serve as a competing endogenous RNA for miR-212-3p to regulate MAPK1. Finally, rescue assays indicated that the inhibitory effect of KCNQ1OT1 knockdown on inflammatory response, cell apoptosis, and p38/NF-κB pathway was reversed by MAPK1 overexpression in HK-2 cells. In conclusion, KCNQ1OT1 aggravates acute kidney injury by activating p38/NF-κB pathway via miR-212-3p/MAPK1 axis in sepsis. Therefore, KCNQ1OT may serve as a potential biomarker for the prognosis and diagnosis of AKI patients.

## Introduction

Sepsis is a systemic inflammatory response syndrome stimulated by infection [1,2]. As a serious complication of sepsis, acute kidney injury (AKI) can result in organ dysfunction and cause high morbidity and mortality in most septic shock patients [3]. Clinically, AKI can be grouped into three primary etiologies: prerenal, renal, and postrenal [4,5]. Various causes of AKI include sepsis, nephrotoxic drugs, dehydration, renal surgery, renal ischemia, ischemia-reperfusion renal injury and urinary tract obstruction [4]. Due to the complexity and uniqueness of sepsis, sepsis-induced AKI is distinct from other types of AKI, which is the first cause of AKI in intensive care unit [6]. Potential pathogenic mechanism underlying sepsis-induced AKI includes cellular metabolic reprogramming, a dysregulated inflammatory response, and microcirculatory dysfunction [7]. At the cellular level, the epithelial-mesenchymal transition (EMT) and the endothelial-to-mesenchymal transition (EndMT) play vital roles in the development of kidney fibrosis [8]. Novel signaling molecules that regulate kidney fibrosis include endothelial glucocorticoid receptors, endothelial Sirtuin 3, endothelial fibroblast growth factor receptor 1 in kidney and podocyte-glucocorticoid receptor signaling in protecting glomerula [9–12]. With the emerging of detection techniques for sepsis-associated AKI, such as evaluation of a urine microscopy score and investigation of novel serum biomarkers, people have more understanding of sepsis-induced AKI [13]. Blood purification and pharmacologic therapies have become promising experimental therapies in recent years [14]. Potential drugs against AKI and renal fibrosis are known as DPP-4 inhibitor linagliptin, empagliflozin, Sirtuin 3, JAK/STAT inhibitors, glycolysis inhibitors, angiotensin-converting enzyme inhibitors, angiotensin receptor antagonists, and peptide AcSDKP [15–21]. However, effective therapeutic strategies for prevention and treatment of sepsis-associated AKI are still limited [14,22]. Therefore, providing new insights into molecular regulatory mechanism associated with AKI is critical to the development of potential therapeutic approaches for AKI.

Many molecules have been reported to be associated with renal injury and fibrosis [23]. For example, SIRT3, an important member of the sirtuin family, can alleviate AKI by improving mitochondrial function and regulating fatty acid oxidation [17,24]. Mitogen-activated protein kinase 1 (MAPK1), also known as p38 or ERK, belongs to the MAP kinase family [25,26]. MAP kinases or ERKs were reported to function as an integration point for several biochemical signaling pathways and involve in various cellular processes like apoptosis, inflammation, and transcription regulation [27–30]. The phosphorylation of upstream kinases could activate MAP kinases. Upon activation, MAP kinase translocates to the nucleus to phosphorylate nuclear targets such as p38 or NF-κB [28,31–33]. This regulatory network of MAPK1 has been identified in various diseases [34–36]. Additionally, the upregulation of miRNA-433-5p activates MAPK1 to protect acute spinal cord injury [37]. MiR-342 suppresses MAPK1 expression to relieve lipopolysaccharide-induced acute lung injury [38]. Guanine-nucleotide exchange factor H1 was proposed to upregulate lipopolysaccharide (LPS)-induced inflammatory cytokines in endothelial cells by activating nuclear factor κB [39]. However, the upstream mechanism of MAPK1 in AKI deserves a further exploration.

MicroRNAs (miRNAs) are highly conserved non-coding transcripts, approximately 18–24 nucleotides in length, which bind to the 3′-UTR of mRNAs to affect their expression in various biological processes [40,41]. Emerging evidence suggests that the alterations of miRNAs can cause fibrotic disorder in kidney [42]. miR200 family and miR205 are highly associated with EMT, and suppression of upregulated miR-21 or overexpression of downregulated miR-125b is a promising approach to inhibit the induction of EndMT [42]. miR-29 family and miR-let-7 family are major anti-fibrotic players in kidney fibrosis [43]. Their participation in crosstalk mechanisms is crucial for endothelial cell homeostasis. miR-29 directly targets DDP-4, and DDP-4 inhibition can suppress EndMT-driven TGFβ signaling in streptozotocin-induced renal fibrosis. Thus, DPP-4 inhibition can be one of therapeutic strategies for diabetic nephropathy [43]. miR-29 and miR-let-7 families exhibit crosstalk regulation by stimulating FGFR1 phosphorylation and targeting TGFβR1 [43]. miR-22-3p targets PTEN to inhibit the inflammatory response and HK-2 cell apoptosis [44]. miR-212-3p has also been documented to restrain inflammatory response by targeting high mobility group box 1 (HMGB1) in LPS-induced murine macrophages [45]. Nevertheless, the interaction between MAPK1 and miR-212-3p is unclear.

Long non-coding RNAs (lncRNAs) are a kind of transcripts with over 200 nucleotides without protein-coding ability [46–48]. LncRNAs can serve as a competing endogenous RNA (ceRNA) to indirectly regulate downstream messenger RNAs by interacting with miRNAs, thus influencing disease phenotypes in kidneys [49]. For example, lncRNA TCONS_00016406 interacts with miR-687 to regulate the expression of phosphatase and tensin homolog (PTEN), thereby attenuating LPS-stimulated AKI, as shown by alleviation of inflammation, the inhibition of oxidative stress and the suppression of cell apoptosis [50]. LncRNA SNHG14 promotes the production of inflammatory cytokines in LPS-induced HK-2 cells by interacting with miR-495-3p to regulate downstream homeodomain interacting protein kinase 1 (HIPK1)/NF-κB/p65 signaling pathway [51]. Potassium voltage-gated channel subfamily Q member 1 opposite strand/antisense transcript 1 (KCNQ1OT1) was reported to exert effects on sepsis-associated diseases including myocardial injury, acute respiratory distress syndrome and preeclampsia in response of LPS treatment [52–54]. Yet, neither the role nor the mechanism of KCNQ1OT1 has been explored in AKI.

The study aimed to explore the role of MAPK1 and its upstream signaling in sepsis-induced AKI. We hypothesized that KCNQ1OT1 might act as a competing endogenous RNA (ceRNA) against miR-212-3p to regulate MAPK1 expression, thereby affecting AKI progression. The study might provide novel biomarkers for the diagnosis and treatment of AKI.

## Materials and methods

### Cell culture

Human renal tubular epithelial cells (HK-2) were purchased from Cell Bank of Type Culture Collection (Chinese Academy of Sciences, Shanghai, China) and were cultured in RPMI-1640 medium (Gibco, New York, CA, USA) containing 100 mg/ml streptomycin, 100 U/ml penicillin and 10% fetal bovine serum (FBS; Gibco) in a humidified atmosphere at 37°C with 5% CO_2_.

### Cell treatment

To establish an *in vitro* model of sepsis, lipopolysaccharide (LPS; 1 μg/ml; Sigma, St. Louis, MO, USA) was used to induce inflammation for 24 h. Cells were then inoculated into 60 mm culture plate (1 × 10^6^ cells/ml) for 16 h of incubation at 37°C. Cells in the control group were treated with the same dose of normal saline (Gibco).

### Cell transfection

Short hairpin RNA (sh-RNA) targeting MAPK1 (sh-MAPK1) or KCNQ1OT1 (sh-KCNQ1OT1) and negative controls (sh-NC), MAPK1 overexpression vector (pcDNA3.1/MAPK1) and the negative control (empty pcDNA3.1 vector), miR-212-3p mimics/inhibitor and the negative control (NC mimics/inhibitor) were obtained from GenePharma (Shanghai, China). Sequences of plasmids used for cell transfection are provided in Table 1. HK-2 cells were seeded in 6-well plates and grown for 24 h until the cell density reached 30–50%. Later, Lipofectamine 2000 (Thermo Fisher Scientific, USA) was used to transfect shRNAs (50 nM), mimics/inhibitors (40 nM) and pcDNA3.1 vectors (10 nM) into HK-2 cells according to the manufacturer’s protocols. After 48 hours, the efficiency of cell transfection was verified by RT-qPCR.

**Table 1.** Sequences of plasmids used for cell transfection

Name	Sequence (5ʹ→3ʹ)
sh-MAPK1	GGACCTCATGGAAACAGATCTTTCAAGAGAAGATCTGTTTCCATGAGGTCCTTTTTT
sh-NC	AGATGACACTATAGGTCCGACTTCAAGAGAGTCGGACCTATAGTGTCATCTTTTTTT
sh-KCNQ1OT1	GGTGTTACGACTTGTTGTATTCAAGAGATACAACAAGTCGTAACACC TTTTTT
sh-NC	GATGTGATCATTCTGGTGTTTCAAGAGAACACCAGAATGATCACATCTTTTTT
miR-212-3p mimics	UAACAGUCUCCAGUCACGGCC
NC mimics	CGCCACCUAAGUAGUGCACCU
miR-212-3p inhibitor	GGCCGUGACUGGAGACUGUUA
NC inhibitor	AGGUGCACUACUUAGGUGGCG

### Animal model

Adult male SpragueDawley (SD) rats (n = 32, 220–250 g) were purchased from Vital River Co. Ltd (Beijing, China). After being anesthetized through intraperitoneal injection of 10% chloral hydrat, rat was cut open on the midline of the abdomen and then separated the mesentery and cecum. Afterward, we ligated the ileocecal valve and punctured the end of cecum twice with needle. The cecum was returned into the abdominal cavity after defecating. In the sham group, the skin was sutured after only separating the mesentery and cecum. The levels of blood urea nitrogen (BUN) and serum creatinine (SCR) were determined by available kits (Thermo Fisher Scientific). After rats were sacrificed 2 weeks after the establishment of the *in vivo* model, blood was collected, and kidneys were isolated for further study. Animal experiments were performed in reference to a previous study [55].

## Preparation and injection of recombinant adeno-associated virus (rAAV)

To manipulate the MAPK1 expression *in vivo*, the rAAV system (serotype 9) were employed [56]. To knockdown MAPK1 expression, the sh-RNA sequence targeting MAPK1 was amplified by PCR, and then ligated into rAAV vectors. Two weeks before animal model establishment, rats were injected with AAV-sh-MAPK1 (1 × 10^12^ copies) or AAV-sh-NC (1 × 10^12^ copies) via tail vein.

## Hematoxylin-eosin (HE) staining

HE staining was conducted as previously described [57]. In short, paraffin-coated renal tissue sections (5 μm) were dewaxed and hydrated with xylene. Next, tissue sections were stained with hematoxylin and eosin solution. After dehydration and sealing, the pathological morphology of renal tissue was visualized by an optical microscope.

## Enzyme-linked immunosorbent assay (ELISA)

As previously reported, 3 mL blood was collected from rats after anesthesia [57]. After blood samples were centrifuged for 10 min at 3500 r/min, concentrations of TNF-α, IL-6 and IL-1β in the serum were examined using ELISA kits (Nanjing Jiancheng Bioengineering Institute, Nanjing, China) according to the manufacturer’s recommendations.

## Flow cytometry analysis

Apoptosis of HK-2 cells was measured utilizing Annexin V-FITC apoptosis detection kit (BD Biosciences; San Jose, CA, USA) according to a previous study [58]. After washing, HK-2 cells were stained by binding buffer including Annexin V-FITC and propidine iodide (PI) in the darkness at 4°C. Afterward, cell apoptosis was evaluated by flow cytometry (FACScan; BD Biosciences, USA). The FITC Annexin V^+^/PI^−^ and FITC Annexin V^+^/PI^+^ cell populations were considered as apoptotic cells.

## Immunofluorescence assay

To explore the nuclear transport level of NF-κB, immunofluorescence assay was performed [59]. HK-2 cells were immobilized with 4% paraformaldehyde. Next, cells were permeabilized through 0.1% Triton X-100 and 0.1% Sodium Citrate. Then, HK-2 cells were incubated with anti-NF-κB (ab32536, 1:250; Abcam, Cambridge, MA, USA) for 2 h following by suitable secondary antibody. DAPI (Sigma-Aldrich) was used for nucleus staining. Finally, the fluorescence microscopy (Olympus America Inc, Center Valley, PA) was used to observe cells.

## Luciferase reporter assay

The full length of KCNQ1OT1 or 3′-UTR of MAPK1 was cloned into the pmirGLO reporters to construct KCNQ1OT1‐wild type (WT) or MAPK1‐WT reporters. The MAPK1‐mutant (Mut) and KCNQ1OT1‐Mut reporters were also established. MiR‐212-3p mimics/inhibitor (or NC mimics/inhibitor) were co-transfected with plasmids mentioned above into HK-2 cells using Lipofectamine 2000 (Thermo Fisher Scientific). After 48 h of transfection, the luciferase activities were examined by Dual-Luciferase Reporter System (Promega, Madison WI, USA). The firefly luciferase activity was normalized to that of Renilla luciferase. The assay was performed according to the previous study [58].

## Reverse transcription quantitative polymerase chain reaction (RT-qPCR)

The RNA from rat renal tissues or HK-2 cells was extracted by TRIzol regent (Invitrogen, USA) and reverse transcribed into cDNAs using PrimeScript RT reagent kits (Takara Biotechnology Ltd., Dalian, China). The SYBR® Premix Ex Taq^TM^ II reagent kit (RR820A, Takara) under ABI7500 real-time qPCR system (7500, ABI Company, Oyster Bay, NY, USA) was used for performing qPCR. Gene expression was calculated by 2^−ΔΔCT^ method [60]. GAPDH was an internal reference for MAPK1 and lncRNAs, and U6 was a control for miRNAs.

## Western blotting

The protein contents were extracted from rat renal tissues or HK-2 cells with RIPA lysate (Beyotime Biotechnology, Shanghai, China). Then, protein samples were separated by 10% SDS-PAGE and moved to polyvinylidene difluoride membranes. After being blocked with skim milk, the membranes were incubated with the primary antibodies at 4°C overnight. Primary antibodies include anti-Bax (ab182733, 1:2000), anti-Bcl-2 (ab182858, 1:2000), anti-cleaved-caspase-3 (ab2302, 1:500), anti-E-cadherin (ab1416, 1:50), anti-collagen I (ab34710, 1:1000), anti-p38MAPK (ab170099, 1:1000), anti-p-p38MAPK (ab178867, 1:1000), anti-NF-κB (ab32536, 1:1000), anti-p-NF-κB (ab76302, 1:1000), and anti-GAPDH (ab9485, 1:2500). Next, secondary antibodies were added to membranes for another 2 h of incubation at room temperature. The bands were assessed through the ECL chemiluminescent detection system (Thermo Fisher Scientific) and quantified by ImageJ software (National Institutes of Health, Bethesda, MA, USA) [61].

## Statistical analysis

All data were shown as mean ± standard deviation (SD) and analyzed by SPSS 19.0 (SPSS, Chicago, IL) [62]. The student’s *t*-test was applied for two-group comparisons, and one-way analysis of variance followed by Tukey’s *post hoc* analysis was applied for multiple-group comparisons. A value of *p* < 0.05 was considered as statistically significant.

## Results

Sepsis-induced AKI is the major cause of AKI in intensive care unit. The exploration of biomarkers for sepsis-associated AKI and their underlying mechanism are necessary for the enrichment of therapeutic approaches. Here, MAPK1 was found to be upregulated in LPS-treated HK-2 cells. MAPK1 knockdown inhibited inflammatory response in LPS-induced HK-2 cells and suppressed cell apoptosis. Additionally, silencing MAPK1 downregulated protein levels of EMT marker (E-cadherin) and mesenchymal marker (collagen I) in LPS-treated HK-2 cells. Moreover, silencing MAPK1 inactivated MAPK/NF-κB pathway in LPS-induced HK-2 cells and alleviated sepsis-induced kidney injury *in vivo*. miR-212-3p was verified to be the upstream molecule of MAPK1. KCNQ1OT1 interacts with miR-212-3p to regulate MAPK1 expression. In rescue experiments, overexpressed MAPK1 reversed the suppressive effect of KCNQ1OT1 knockdown on HK-2 cell injury and p38/NF-κB signaling. Overall, KCNQ1OT1 aggravates sepsis-induced AKI by activating p38/NF-κB signaling via miR-212-3p/MAPK1 axis.

## Knockdown of MAPK1 suppresses inflammatory response in LPS-induced HK-2 cells

**Figure 1.**
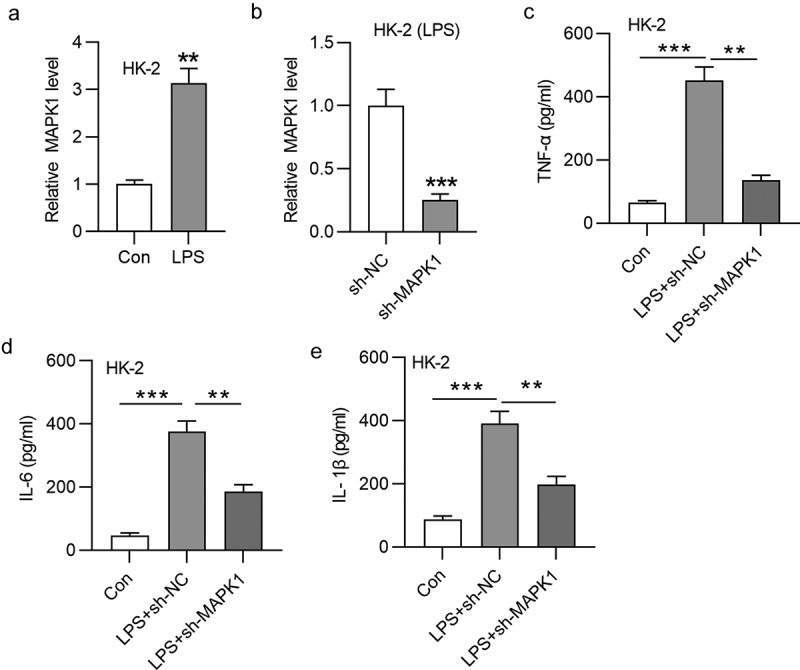
MAPK1 knockdown suppresses inflammatory response in LPS-induced HK-2 cells

We first focused on the role of MAPK1 in sepsis-induced AKI. According to RT-qPCR, MAPK1 level was increased by LPS treatment in HK-2 cells (Figure 1a). The knockdown efficiency of the plasmid sh-MAPK1 is confirmed in Figure 1b. Next, through ELISA, the increase in TNF-α, IL-6 and IL-1β concentration was discovered in response of LPS treatment, which was counteracted by silencing MAPK1 (Figure 1c–e). In general, MAPK1 knockdown inhibits inflammatory response in LPS-induced HK-2 cells.

(A) RT-qPCR was performed to examine mRNA expression of MAPK1 in HK-2 cells treated with or without LPS. (B) To detect the knockdown efficiency of MAPK1, MAPK1 expression in HK-2 cells transfected with sh-NC or sh-MAPK1 was evaluated by RT-qPCR. (C-E) The concentration of TNF-α, IL-6 and IL-1β in three groups: HK-2 cells, HK-2 cells treated with LPS and transfected with sh-NC, and HK-2 cells treated with LPS and transfected with sh-MAPK1 was assessed by ELISA. **p < 0.01 and ***p < 0.001.

## Silencing MAPK1 inhibits HK-2 cell apoptosis and inactivates p38/NF-κB signaling pathway

**Figure 2.**
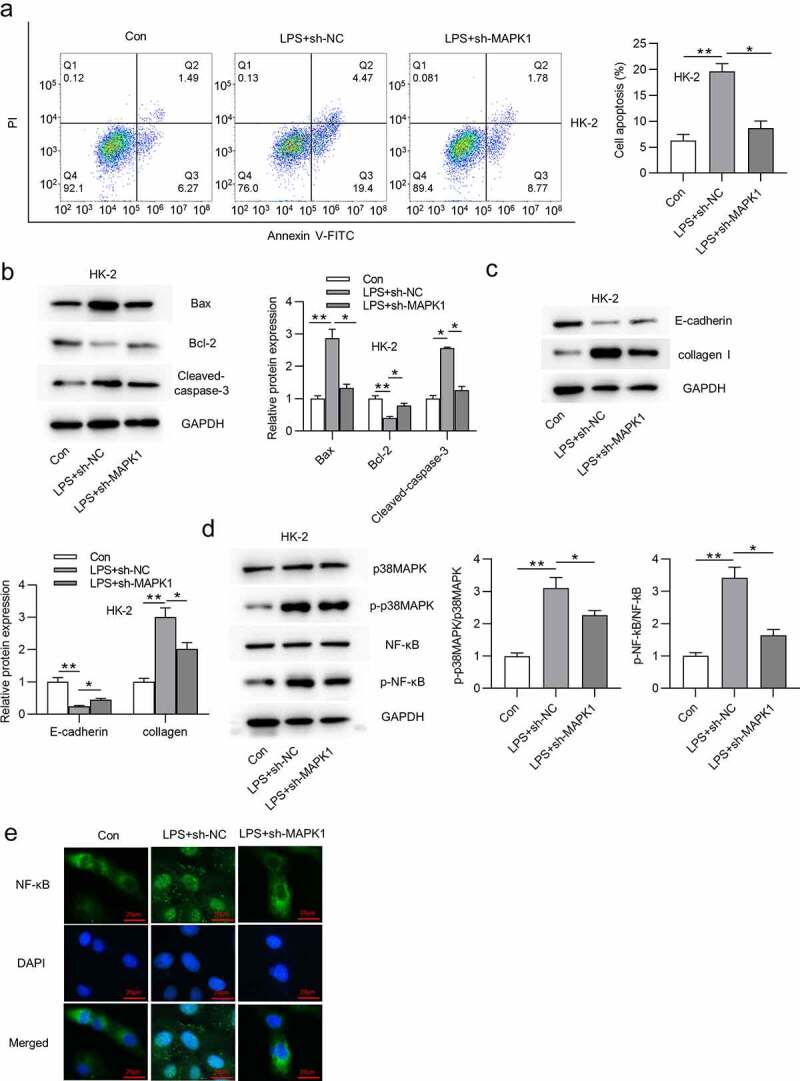
Silencing MAPK1 inhibits HK-2 cell apoptosis and inactivates p38/NF-κB pathway

Subsequent work was done to explore the effect of MAPK1 on cell apoptosis. As displayed in Figure 2a, LPS treatment significantly enhanced the apoptosis of HK-2 cells, but this change could be reversed after silencing MAPK1. Western blotting was performed to examine protein levels of apoptosis markers (Bax, Bcl-2, and cleaved caspase-3), EMT marker (E-cadherin), and fibrosis marker (collagen I) in HK-2 cells with indicated transfection. The results demonstrated that the LPS treatment decreased Bcl-2 protein level but increased Bax and Cleaved caspase-3 protein levels (Figure 2b). Moreover, protein level of E-cadherin was reduced and that of collagen I was increased in HK-2 cells stimulated by LPS (Figure 2c). All these alterations were reversed by MAPK1 knockdown (Figure 2b–c). MAPK1 is involved in the activation of many pathways, among which p38/NF-κB pathway has been reported to be associated with cell apoptosis and inflammatory response. Western blotting revealed that p-p38MAPK and p-NF-κB protein levels were enhanced in response of LPS treatment and then reduced in LPS-treated HK-2 cells transfected with sh-MAPK1 (Figure 2d). In addition, knockdown of MAPK1 also repressed the nuclear translocation of NF-κB induced by LPS treatment (Figure 2e). To sum up, silencing MAPK1 inhibits HK-2 cell apoptosis and inactivates p38/NF-κB pathway.

(A) The apoptosis rate of HK-2 cells in three groups (control group, LPS+sh-NC group, and LPS+sh-MAPK1 group) was measured by flow cytometry analysis. (B-C) Protein levels of apoptotic markers (Bax, Bcl-2 and Cleaved caspase-3), EMT marker (E-cadherin), and fibrosis marker (collagen I) in HK-2 cells from above three groups were quantified by Western blotting. (D) Protein levels of p38MAPK, phosphorylated p38MAPK, NF-κB and phosphorylated NF-κB in above three groups were detected via Western blotting. (E) The nuclear translocation of NF-κB in above three groups was assessed by immunofluorescence assay. *p < 0.05 and **p < 0.01.

## MAPK1 depletion alleviates sepsis-induced kidney injury *in vivo*

**Figure 3.**
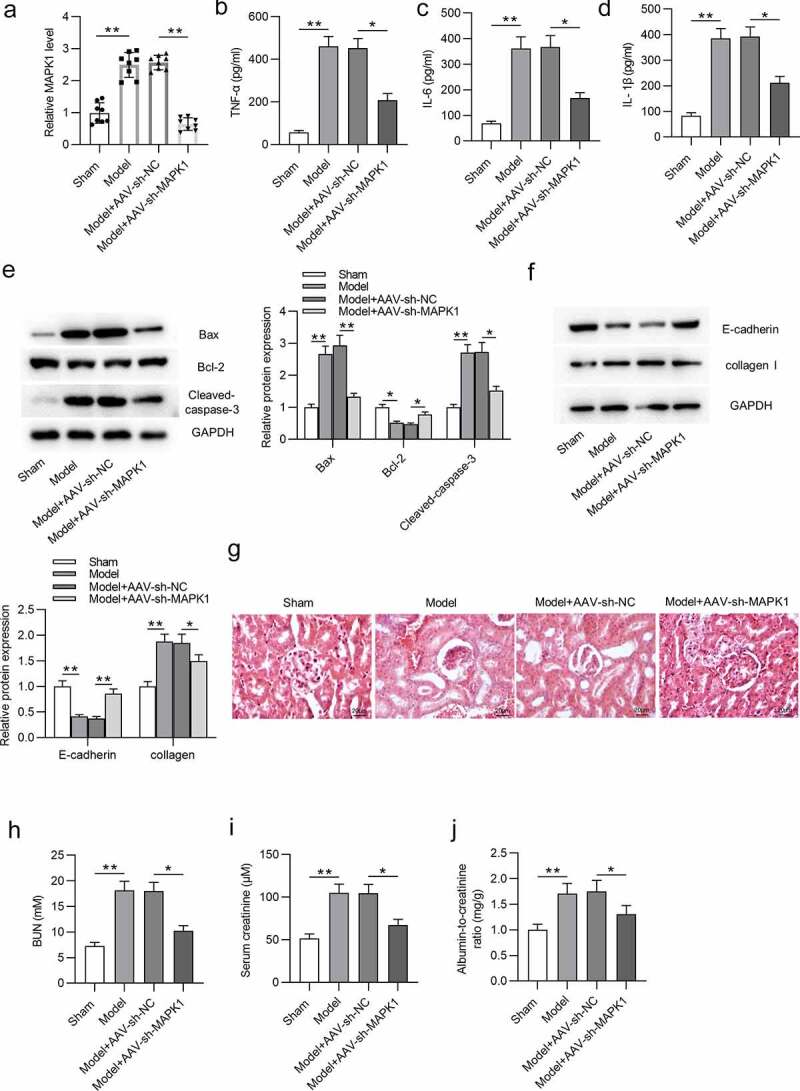
MAPK1 depletion alleviates sepsis-induced kidney injury *in vivo.*

We then investigated the effect of MAPK1 on sepsis-induced AKI *in vivo*. First, the knockdown efficiency of MAPK1 is ensured in Figure 3a. The concentration of proinflammatory cytokines (TNF-α, IL-6 and IL-1β) was reduced by MAPK1 inhibition in model rats (Figure 3b–d). After silencing MAPK1, Bax and Cleaved caspase-3 protein levels were reduced while Bcl-2 protein level was enhanced *in vivo* compared with these in the Model+AAV-Sh-NC group (Figure 3e). Additionally, E-cadherin protein level was reduced while collagen I level was increased in Model group compared with those in sham group, and MAPK1 depletion partially reversed the alternations (figure 3f). The distorted renal tubules, apoptotic or/and necrotic cell phenotypes were observed in Model group, but MAPK1 repression alleviated these effects (Figure 3g). In addition, serum creatinine levels in Model group were increased from 0.93 mg/dL to 1.89 mg/dL compared with those in the sham group, which indicated the successful establishment of the model of sepsis-induced AKI (Figure 3i). Moreover, increased levels of BUN, serum creatinine, and albumin-to-creatinine ratio in Model group were retarded after MAPK1 inhibition (Figure 3h–j). The These data suggested that MAPK1 deficiency alleviates sepsis-induced kidney injury *in vivo*.

(a) The knockdown efficiency of AAV-sh-MAPK1 was assessed by RT-qPCR in four groups (n = 8 rats/group): sham group, Model group, Model+AAV-sh-NC group, and Model+AAV-sh-MAPK1 group. (B-D) The concentration of TNF-α, IL-6 and IL-1β in rat renal tissues collected from above four groups (n = 8 rats/group) was examined using ELISA. (E-F) The protein levels of apoptotic markers (Bax, Bcl-2 and Cleaved caspase-3), EMT marker (E-cadherin), and fibrosis marker (collagen I) in indicated groups (n = 8 rats/group) were detected by Western blotting. (G) HE staining was used to analyze the histological changes in kidney of rats from four groups (n = 8 rats/group). (H-J) The BUN and serum creatinine levels as well as albumin-to-creatinine ratio in kidney tissues were measured in indicated groups (n = 8 rats/group). *p < 0.05 and **p < 0.01.

## MiR-212-3p directly targets MAPK1

**Figure 4.**
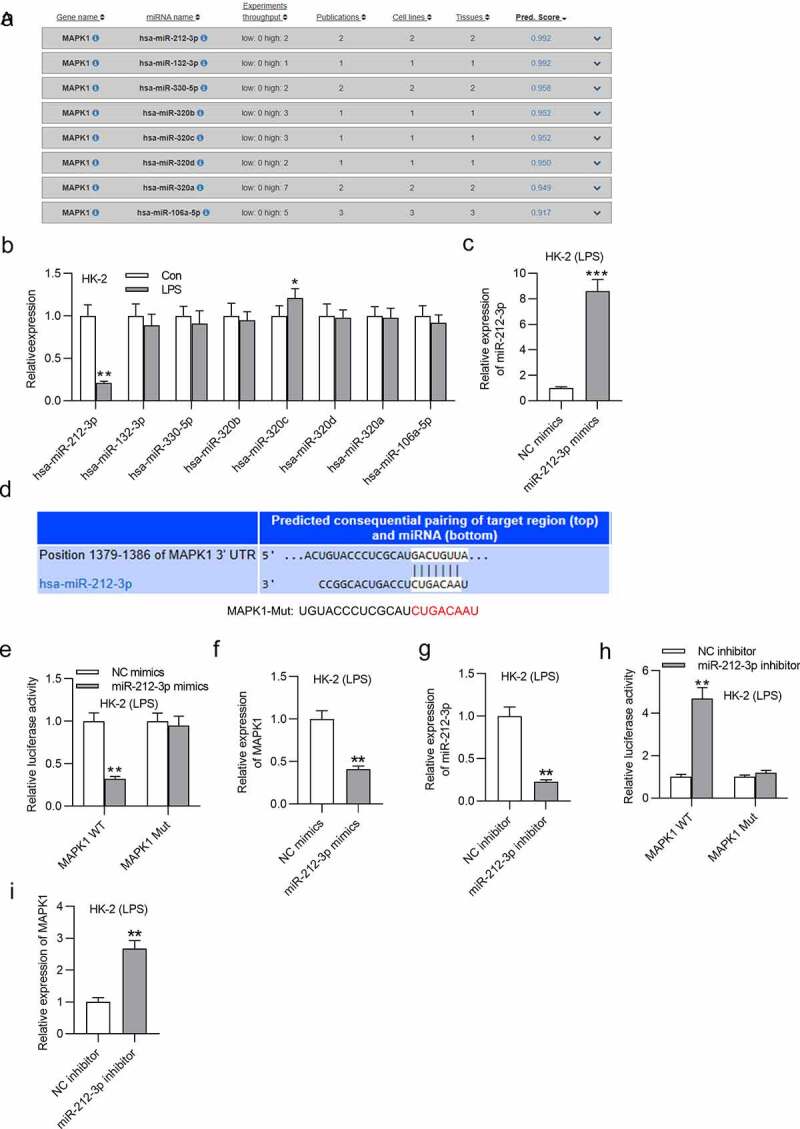
MiR-212-3p directly targets MAPK1

DIANA website (http://carolina.imis.athena-innovation.gr/diana_tools/web/index.php?r=site%2Findex) was applied to search miRNAs that bind with MAPK1 with the screening condition of predicted score > 0.9. Eight candidate miRNAs are displayed in Figure 4a. Among these predicted miRNAs, miR-212-3p exhibited significantly low expression after LPS treatment (Figure 4b). Hence, miR-212-3p was selected for following study. The miR-212-3p level was effectively overexpressed by miR-212-3p mimics (Figure 4c). Then, the binding site between miR-212-3p and MAPK1 was predicted by Targetscan (http://www.targetscan.org/vert_72/) and was shown in Figure 4d. Additionally, the luciferase activity of MAPK1-WT reporters was damaged by miR-212-3p overexpression, but that of MAPK1-Mut reporters were not significantly changed (Figure 4e). Similarly, MAPK1 expression was also reduced by overexpressing miR-212-3p in LPS-induced HK-2 cells (figure 4f). Next, miR-212-3p expression was silenced in LPS-treated HK-2 cells. RT-qPCR revealed a significant decrease in miR-212-3p expression after transfection of miR-212-3p inhibitor (Figure 4g). The luciferase activity of MAPK1 WT was increased in LPS-induced HK-2 cells transfected with miR-212-3p inhibitor (Figure 4h). In addition, mRNA expression of MAPK1 was upregulated by silenced miR-212-3p in LPS-treated HK-2 cells (Figure 4i). In summary, miR-212-3p directly targets MAPK1 3ʹ-UTR and negatively regulates MAPK1 in LPS-induced HK-2 cells.

(a)DIANA website was used for predicting the miRNAs that possess binding site with MAPK1. (B) The expression of candidate miRNAs (miR-212-3p, miR-132-3p, miR-330-5p, miR-320b, miR-320 c, miR-320d, miR-320a and miR-106-5p) in HK-2 cells treated with or without LPS was measured with RT-qPCR. (C) The overexpression efficiency of miR-212-3p was determined by RT-qPCR in LPS-treated HK-2 cells. (D-E) The binding ability between miR-212-3p and MAPK1 was measured by luciferase reporter assay. (F) The MAPK1 expression in response of miR-212-3p mimics was tested by RT-qPCR in LPS-stimulated HK-2 cells. (G) The knockdown efficiency of miR-212-3p inhibitor in cells was determined by RT-qPCR. (H) The luciferase activity of MAPK1 WT/Mut in LPS-induced HK-2 cells transfected with miR-212-3p inhibitor or NC inhibitor was determined by luciferase reporter assay. (I) The impact of miR-212-3p inhibitor on MAPK1 mRNA expression was examined by RT-qPCR. *p < 0.05, **p < 0.01 and ***p < 0.001.

## KCNQ1OT1 directly interacts with miR-212-3p

**Figure 5.**
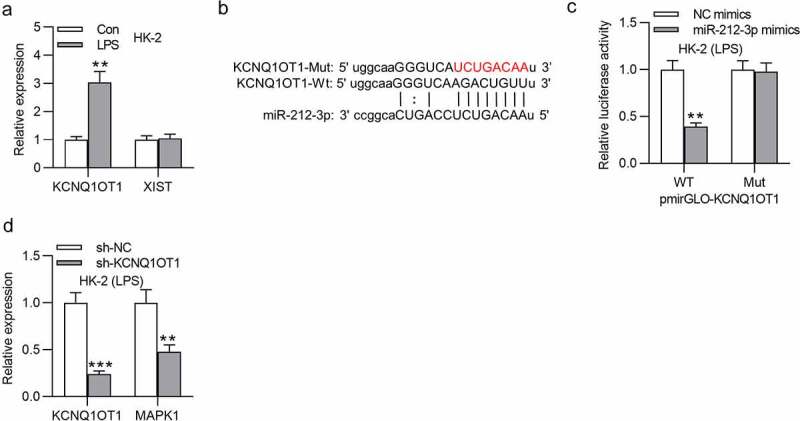
KCNQ1OT1 directly interacts with miR-212-3p

Many studies have reported that lncRNAs bind to miRNAs as a ceRNA and thus release mRNAs, and this ceRNA regulatory network is involved in the development of various human diseases [63,64]. Through the starBase website (http://starbase.sysu.edu.cn/), several lncRNAs are predicted to have binding site for miR-212-3p (Table S1). Particularly, KCNQ1OT1 and XIST possess more binding site on miR-21-3p than other candidate lncRNAs. RT-qPCR revealed that KCNQ1OT1 level was upregulated in the LPS group while XIST expression showed no significant changes (Figure 5a). The binding site between miR-212-3p and KCNQ1OT1 as well as the mutant sequence of KCNQ1OT1 were shown in Figure 5b. The luciferase activity of KCNQ1OT1-WT reporters was reduced by miR-212-3p mimics while that of KCNQ1OT1-Mut was not significantly affected (Figure 5c). KCNQ1OT1 and MAPK1 levels were both reduced by knockdown KCNQ1OT1 (Figure 5d). In conclusion, KCNQ1OT1 directly interacts with miR-212-3p.

(a)KCNQ1OT1 and XIST expression levels in HK-2 cells treated with or without LPS were examined by RT-qPCR. (B-C) The binding ability between KCNQ1OT1 and miR-212-3p was determined via luciferase reporter assay. (D) The KCNQ1OT1 and MAPK1 expression levels in the context of KCNQ1OT1 knockdown was evaluated by RT-qPCR. **p < 0.01 and ***p < 0.001.

## KCNQ1OT1 promotes inflammatory response in LPS-treated HK-2 cells via upregulation of MAPK1

**Figure 6.**
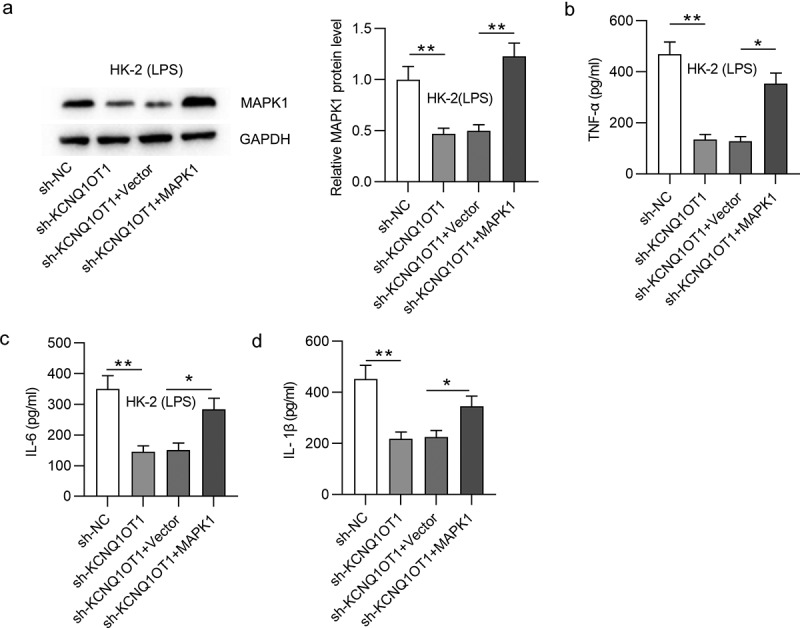
KCNQ1OT1 promotes inflammatory response in LPS-treated HK-2 cells via upregulation of MAPK1

As shown in Figure 6a, inhibition of KCNQ1OT1 decreased MAPK1 protein level, and this effect was rescued after transfection of pcDNA3.1/MAPK1. Furthermore, the decreased content of TNF-α, IL-6 and IL-1β caused by KCNQ1OT1 depletion could be reversed by MAPK1 overexpression (Figure 6b–d). These findings indicated that KCNQ1OT1 facilitates inflammatory response via upregulation of MAPK1.

(a)The protein level of MAPK1 in LPS-treated HK-2 cells from four groups (sh-NC, sh-KCNQ1OT1, sh-KCNQ1OT1+ vector, and sh-KCNQ1OT1+ MAPK1) was measured by Western blotting. (B-D) The concentration of TNF-α, IL-6 and IL-1β was tested by ELISA in LPS-treated HK-2 cells from above four groups. *p < 0.05 and **p < 0.01.

## KCNQ1OT1 promotes HK-2 cell apoptosis and activates p38/NF-κB pathway via upregulation of MAPK1

**Figure 7.**
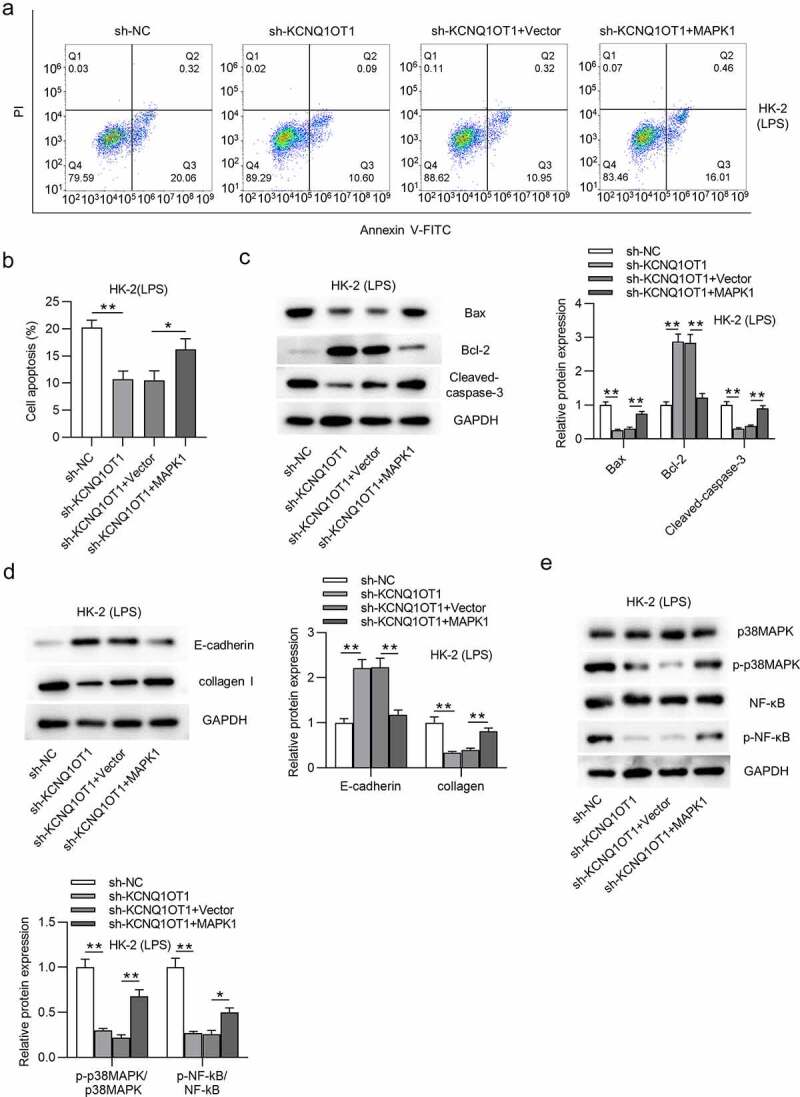
KCNQ1OT1 promotes HK-2 cell apoptosis and activates p38/NF-κB pathway in LPS-treated HK-2 cells via upregulation of MAPK1

Finally, we explored whether KCNQ1OT1 promotes HK-2 cell apoptosis and activates MAPK/NF-κB pathway by upregulation of MAPK1. Overexpression of MAPK1 counteracted the inhibitory effect on cell apoptosis caused by silencing KCNQ1OT1 (Figure 7a–b). The decrease in Bax and cleaved-caspase-3 protein levels and the increase in Bcl-2 protein level induced by silenced KCNQ1OT1 were reversed by MAPK1 up-regulation (Figure 7c). Additionally, MAPK1 overexpression reversed the enhancement of E-cadherin protein level and the reduction of collagen I protein level in LPS-treated HK-2 cells (Figure 7d). p-p38MAPK and p-NF-κB protein levels were reduced by suppressing KCNQ1OT1, but overexpressed MAPK1 neutralized this inhibitory effect (Figure 7e). These results revealed that KCNQ1OT1 promotes cell apoptosis and activates p38/NF-κB pathway via upregulation of MAPK1.

(A-B) The cell apoptosis rate was evaluated using flow cytometry analysis in four groups (sh-NC, sh-KCNQ1OT1, sh-KCNQ1OT1+ vector, and sh-KCNQ1OT1+ MAPK1). (C-D) The protein levels of apoptotic markers (Bax, Bcl-2 and Cleaved caspase-3), EMT marker (E-cadherin), and fibrosis marker (collagen I) were detected by Western blotting in above four groups. (E) The p38MAPK, p-p38MAPK, NF-κB and p-NF-κB protein levels were detected by Western blotting in above four groups. *p < 0.05 and **p < 0.01.

## Discussion

AKI is a heterogeneous group of conditions characterized by a sudden decrease in glomerular filtration rate (GFR) and then an increase in serum creatinine concentration (SCC) or oliguria [65]. Clinically, three etiologies of AKI are prerenal, renal and postrenal one. Prerenal azotemia is featured with the decrease in GFR because of a decrease in renal perfusion pressure with little damage to renal parenchyma [5]. Postrenal etiology of AKI is acute urinary tract obstruction which increases intratubular pressure and thereby decreases GFR [5]. Renal causes of AKI are difficult to define, but evaluating damage to the tubules, glomeruli, interstitium and intrarenal blood vessels can be helpful for considering etiologies of intrinsic renal failure [5]. In the present study, molecules involved in sepsis-induced AKI were investigated.

MAPK1 has been researched to be involved in the regulation of many diseases [37,38,66]. However, the role of MAPK1 in sepsis-induced AKI remains unknown. MAPKs belong to the serine/threonine protein kinase family, and activated MAPKs stimulated cellular responses [67,68]. In this work, MAPK1 expression was upregulated in LPS-treated HK-2 cells. Silencing MAPK1 inhibited LPS-stimulated inflammatory response, HK-2 cell apoptosis, and EMT process. Additionally, MAPK1 depletion suppressed the protein level of renal fibrosis marker (collagen I) compared with the LPS group. NF-κB was reported to be a downstream target of p38-MAPK. Activation of p38-MAPK leads to phosphorylation of I-κB, which causes degradation of I-κB. NF-κB is depolymerized with I-κB and then activates nuclear translocation [69,70]. p38/NF-κB pathway plays an important role in many inflammatory diseases [70–72]. In our study, silencing MAPK1 inactivated the p38/NF-κB pathway in LPS-induced HK-2 cells. Moreover, the impact of MAPK1 on sepsis-induced kidney injury *in vivo* was also revealed. After establishment of *in vivo* model of sepsis, serum creatinine levels show an average increase of 0.96 mg/dL in the model group. According to the criteria for the diagnosis of AKI, i.e., either 25% to 50% increase of baseline serum creatinine levels and/or an absolute elevation of 0.5–2.0 mg/dL from baseline, the results suggested that the establishment of sepsis-induced AKI model was achieved. The criterion was stipulated by the Acute Dialysis Quality Initiative (ADQI) proposing the RIFLE (Risk, Injury, Failure, Loss and End-stage kidney disease) system and Acute Kidney Injury Network (AKIN) [73]. We found that silencing MAPK1 alleviated kidney injury *in vivo*, as shown by alleviation of inflammatory response, inhibition on cell apoptosis, and the decrease in levels of renal injury indicators (BUN, serum creatine) and albumin-to-creatinine ratio.

MiRNAs has been claimed to bind with mRNAs to promote degradation or inhibit translation [74–76]. For example, miR-211 is downregulated in HK-2 cells under hypoxia-reoxygenation condition, which inhibits HK-2 cell apoptosis and alleviates ischemia/reperfusion-induced kidney injury by targeting TGFβR2/TGF-β/SMAD3 signals [77]. Herein, miR-212-3p was predicted and confirmed to directly target MAPK1 3ʹ-UTR and negatively regulate MAPK1 expression. miR-212-3p was downregulated in LPS-treated HK-2 cells. miR-212-3p directly targeted 3ʹ-UTR of MAPK1 and negatively regulated MAPK1 expression in cells. Previously, miR-212-3p regulates HMGB1 to weaken LPS-induced inflammatory response in murine macrophages [45]. Moreover, miR-212-3p decreased SOX5 expression to retard proliferation and strengthen apoptosis of fibroblast-like synoviocytes in rheumatoid arthritis [78]. Hence, we hypothesized that miR-212-3p probably exert an anti-inflammatory effect on LPS-induced HK-2 cells.

Emerging studies proposed that MAPK1 was also regulated by certain lncRNAs in a competitive endogenous RNA (ceRNA) pattern [79,80]. For example, lncRNA metastasis associated lung adenocarcinoma transcript 1 (MALAT1) aggravates coronary atherosclerotic heart disease by interacting with miR-15b-5p to regulate MAPK1 and thus activating mTOR signaling pathway [79]. Moreover, lncRNA taurine upregulated 1 (TUG1) was reported to exert a protective effect on lipopolysaccharide‑induced podocyte injury by competing with MAPK1 to interact with miR-197 [80]. In our study, KCNQ1OT1 was validated to interact with miR-212-3p to upregulate MAPK1. According to previous studies, the role of KCNQ1OT1 is controversial in sepsis-associated diseases. In detail, KCNQ1OT1 displays low level in cardiac tissues of septic rats and alleviates myocardial injury [54]. However, another study proposed that KCNQ1OT1 functioned as an activator of inflammatory response by modulating neutrophil extracellular trap formation in LPS-induced acute respiratory distress syndrome [53]. Results from our work indicated that silencing KCNQ1OT1 restrained inflammatory response in LPS-stimulated HK-2 cells and inhibited cell apoptosis, and these results were reversed by MAPK1 overexpression. Additionally, KCNQ1OT1 was reported to participate in the activation of p38/NF-κB pathway in pituitary adenomas and acute myocardial injury [81,82]. Similarly, in our study, knockdown of KCNQ1OT1 inhibited the phosphorylation of p38 and NF-κB. However, MAPK1 overexpression reversed the suppressive effect of silenced KCNQ1OT1 on protein levels of phosphorylated p38MAPK and NF-κB. The results suggested that KCNQ1OT1 activates p38/NF-κB signaling by upregulating MAPK1.

In summary, KCNQ1OT1 aggravates sepsis-induced AKI by activating p38/NF-κB pathway via miR-212-3p/MAPK1 axis. This work may provide a promising biomarker for the treatment of sepsis-induced AKI. To some extent, there are limitations in our work. For example, the specific mechanism of LPS treatment inducing the dysregulation of KCNQ1OT1, miR-212-3p and MAPK1 in HK-2 cells remains unclear. In addition, the role of KCNQ1OT1 and miR-212-3p in sepsis-induced AKI needs to be further explored in the future.

## Conclusion

KCNQ1OT1 is upregulated in LPS-stimulated HK-2 cells. KCNQ1OT1 deficiency inhibits inflammatory response in LPS-induced HK-2 cells and suppresses EMT, EndMT and cell apoptosis. KCNQ1OT1 interacts with miR-212-3p to regulate the expression of MAPK1. MAPK1 3ʹ-UTR is directly targeted by miR-212-3p. KCNQ1OT1 activates the p38/NF-κB signaling by upregulating MAPK1. MAPK1 depletion alleviates sepsis-induced kidney injury *in vivo*. In conclusion, KCNQ1OT1 competes with MAPK1 for the opportunity of interacting with miR-212-3p and release MAPK1 to activate p38/NF-κB signaling, thus aggravating sepsis-induced AKI.

## Data Availability

The datasets used during the current study are available from the corresponding author on reasonable request.
